# Development of a Sensitive and Specific Novel qPCR Assay for Simultaneous Detection and Differentiation of Mucormycosis and Aspergillosis by Melting Curve Analysis

**DOI:** 10.3389/ffunb.2021.800898

**Published:** 2022-01-24

**Authors:** Mragnayani Pandey, Immaculata Xess, Janya Sachdev, Usha Yadav, Gagandeep Singh, Dibyabhaba Pradhan, Ashit Bhushan Xess, Bhaskar Rana, Lalit Dar, Sameer Bakhshi, Rachna Seth, Manoranjan Mahapatra, Viveka P. Jyotsna, Arun Kumar Jain, Rakesh Kumar, Reshu Agarwal, Prashant Mani

**Affiliations:** ^1^Department of Microbiology, All India Institute of Medical Sciences, New Delhi, India; ^2^ICMR Computational Genomics Centre Informatics, Systems & Research Management Division Indian Council of Medical Research, All India Institute of Medical Sciences, New Delhi, India; ^3^Department of Medical Oncology, All India Institute of Medical Sciences, New Delhi, India; ^4^Department of Pediatrics, All India Institute of Medical Sciences, New Delhi, India; ^5^Department of Hematology, All India Institute of Medical Sciences, New Delhi, India; ^6^Department of Endocrinology, All India Institute of Medical Sciences, New Delhi, India; ^7^Departments of Environmental Toxicology and Bioinformatics, ICMR-National Institute of Pathology Sri Ramachari Bhawan, Safdarjang Hospital Campus, New Delhi, India; ^8^Department of Otorhinolaryngology, All India Institute of Medical Sciences, New Delhi, India

**Keywords:** invasive mucormycosis, invasive aspergillosis, real-time PCR, melting curve analysis, invasive fusariosis

## Abstract

Molecular diagnostic assays can expedite the diagnosis of fungal infections, and subsequently help in early interventions and appropriate management of patients. The aim of this study was to develop a single set of primers for a real-time quantitative polymerase chain reaction (qPCR) assay to detect and identify commonly reported, clinically relevant molds i.e., *Aspergillus spp, Mucorales* and *Fusarium spp.*, up to genus level by melting curve analysis. This assay was evaluated in whole blood from patients with suspected invasive aspergillosis (IA), and in tissue biopsy, bronchoalveolar lavage (BAL) fluid and other site-specific samples from patients with suspected invasive mucormycosis (IM). The limit of detection (LoD) was determined as 10 copies/μl for all three molds. The mean coefficient of variation (CV) across all sets of intra- and inter-assay data was 0.63% (ranging from 0.42 to 1.56%), showing high reproducibility of the assay. Sensitivity and specificity of the assay were 93.3 and 97.1% respectively for diagnosis of IA, and 99.29 and 83.84% respectively for diagnosis of IM. *Fusarium* was not detected in any of the clinical samples included and the few laboratory confirmed cases of fusariosis did not meet the inclusion criteria of the study. Hence no ROC curve or cutoff value could be generated for the same. This newly developed qPCR assay therefore appears to be a promising tool in detection of IA and IM.

## Introduction

Limitations in conventional diagnostic methods often lead to delays in diagnosis of invasive fungal infections (IFIs), which in turn delays treatment and is thus a key risk factor for poor patient outcomes (Barnes, 2008). Early and accurate diagnosis of IFIs, is therefore essential, and can be achieved by molecular methods, which also require lesser mycology-specific diagnostic experience and smaller sample quantities than conventional diagnostic methods.

Several conventional, real-time and nested PCR assays have been designed for the specific detection of single or multiple fungal pathogens (Kami et al., 2001; Cornet et al., 2002; Costa et al., 2002; Sanguinetti et al., 2003; Simoneau et al., 2005; Meersseman et al., 2007; Botterel et al., 2008; Imbert et al., 2016, 2018). Unlike conventional PCR, real-time PCR techniques can quantify the load of fungal DNA in a sample and hence can also distinguish between fungal colonizers and invasive pathogens (Li et al., 2016; Valero et al., 2016).

A range of target genes have been used in development of quantitative PCR (qPCR) assays, but over the last few years the ribosomal DNA gene region (consisting of the 18 S, 5.8 S, and 28 S domains, separated by the ITS1 and ITS2 regions), has been shown to be a promising target (Musher et al., 2004; White et al., 2006; Springer et al., 2011; Valero et al., 2016). Numerous studies have used target sequences from this region to detect fungal DNA (Sugita et al., 2004; Springer et al., 2016). There are a variety of commercially available PCR assays currently in use, but only a few have been investigated in large cohort studies (e.g., AsperGenius®, MycAssay Aspergillus®, MycoGENIE®) (Guinea et al., 2013; White et al., 2015; Dannaoui et al., 2017; Salzer et al., 2019).

Most studies have been focused either on *Aspergillus spp or Mucorales*; these are most useful in confirmatory diagnosis of cases where signs and symptoms are specific and suggestive of a particular IFI. More often, there is need for an assay which can detect multiple pathogens in a single reaction, but combined studies on both these groups of medically important fungi are scant in existing literature. Melting curve analysis has been reported by several authors as a fast, reliable and cost-effective method for identification of multiple fungal species using a single PCR assay, while avoiding use of multiple expensive TaqMan probes, as well as the additional financial cost and longer turnaround time of amplicon sequencing (Arancia et al., 2011; Alonso et al., 2012; Lengerova et al., 2014).

To address the urgent clinical need for a single sensitive and specific molecular assay which can detect and identify multiple fungal pathogens and also provide an estimate of fungal load, we have developed a mold-specific quantitative real-time PCR assay, permitting the detection and differentiation of three clinically relevant molds (*Aspergillus spp. Mucorales* and *Fusarium spp*.) in a single reaction, using one set of primers and melting curve analysis. In this study, we describe the technical features of the assay and assess its potential benefits by evaluation using clinical samples.

### Methodology

#### Primer Design

A total of three mold-specific primer sets were designed using HYDEN and Primer 3 software. The complete sequences of the ribosomal region (including 18 s, 28 s 5 s and ITS) of the genomes of 16 species from all three targeted mold groups −7 Aspergillus spp (*A. flavus, A. fumigatus, A. niger, A. nidulans, A. sydowii, A. lentulus, A. glaucus*); 7 *Mucorales* (*Rhizopus arrhizus, Rhizopus microsporus, Lichtheimia corymbifera, Apophysomyces variabilis, Mucor circinelloides, Rhizomucor pusillus, Cunninghamella bertholletiae*) and 2 *Fusarium spp (F. solani, F. oxysporum—*were downloaded from the National Center for Biotechnology Information (NCBI) public genetic database (GenBank). Alignment of sequences was done using Clustal X software and revealed conserved regions with some differences. These were the basis for selection of primers that annealed at 60°C to target sequences of the three targeted molds, but not other unrelated fungal genera. While the primers used in this assay able to detect dermatophytes, this aspect was not analyzed further as the focus of the study was on invasive fungal infections, not superficial mycosis.

Furthermore, this primer amplifies a larger fragment of dermatophytes (~472 bp) that can easily be distinguished from other clinically significant molds such as *Mucorales, Aspergillus, and Fusarium*, the product size of which is <225 bp. Although it was not tested further in real-time PCR, online software (https://dna-utah.org/tm/) was used to calculate the melting temperature of the product, which was found to be ~95°C. As a result, it can be distinguished from the three target molds using melting curve analysis.

**Figure 1:**
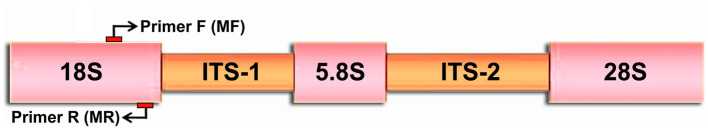
Schematic diagram of rDNA showing Forward (MF) and Reverse primer (MR) annealing positions. The arrows show the annealing position for primers in the 18S ribosomal subunit.

Primer specificity for the targeted molds was evaluated using DNA extracted from clinical isolates mentioned in Supplementary Table 1, among which all *Aspergillus spp, Mucorales* and *Fusarium spp* were identified to species level by Sanger sequencing of ITS region (accession numbers for all the isolates have been provided in Supplementary Table 2), and other isolates were identified by conventional lactophenol cotton blue mount (Supplementary Tables 1, 2). Finally, one degenerate primer set M1F (5′-ACCTTGRCCGRRAGGTCYGGGT-3′) and M1R (5′-CMSKRRRKGRYYGTTGCC-3′) with the highest specificity was selected for study. This primer set amplifies a fragment (223 bp in *Mucorales*, 223 bp in *Aspergillus spp*. and 222 bp in *Fusarium spp*.) of the gene coding for the small ribosomal subunit (18S rRNA) (Figure 1). Agarose gel electrophoresis image of PCR products of newly designed mold specific primer set is given in Supplementary Figure 1.

The selected primers were subjected to a BLAST search within the GenBank sequence database (http://blast.ncbi.nlm.nih.gov/Blast.cgi) to avoid cross-homology with other unrelated micro-organisms.

### Standardization of Real Time Quantitative PCR Using Designed Primers

#### Preparation of Standards for Quantitative Real-Time PCR

Initially, DNA extracted from all 16 molds used in primer design was subjected to the qPCR assay with the final primer set. However, within each mold group, the sizes and sequences of PCR products were consistent across species, as was the melting temperature-−85.5°C for all 7 *Aspergillus spp*., 83.5°C for all 7 *Mucorales*, and 87°C for both *Fusarium spp*. Therefore the PCR product of only one species from each group was selected for preparation of the standard—*Aspergillus flavus* from the *Aspergillus spp, Rhizopus arrhizus* from *Mucorales*, and *Fusarium solani* from the *Fusarium spp*. The PCR products for these three molds were cloned in a TOPO-TA vector and concentration of each was spectrophotometrically determined. The plasmid copy number for each cloned product was calculated using online software at the link https://cels.uri.edu/gsc/cndna.html. Serial dilutions of each plasmid product ranging from 10^9^ to 10 copies/μl were prepared in TE buffer, to be used as standards. These standards were subjected to real time PCR amplification, and the results were used in preparation of a standard curve and melting curve analysis.

#### PCR Amplification Conditions

The PCR was conducted in an Applied Bio Systems StepOne Plus PCR machine (Model No 2.72E+08), Each well contained a final reaction volume of 20 μl, consisting of 10 μl of 2X qPCR master mix (GCC Biotech Pvt. Ltd, India), 2 μl of 10X reporter dye (ROX + SYBR Green) 1 μl of each primer, 1 μl of standard/control strain DNA/sample DNA, and 6.6 μl of nuclease free water. PCR conditions were as follows: an initial heating step for 2 mins at 95°C, followed by 40 cycles of amplification consisting of denaturation at 95°C for 30 secs, annealing at 60°C for 60 secs, and extension at 72°C for 30 secs. Melting curves of amplicons were analyzed by measuring the fluorescence of SYBR green dye at different temperatures (from 65 to 99°C) Melting curves were automatically displayed as a graphical chart from which melting temperatures were recorded.

#### Standardization of the Assay

**Figure 2:**
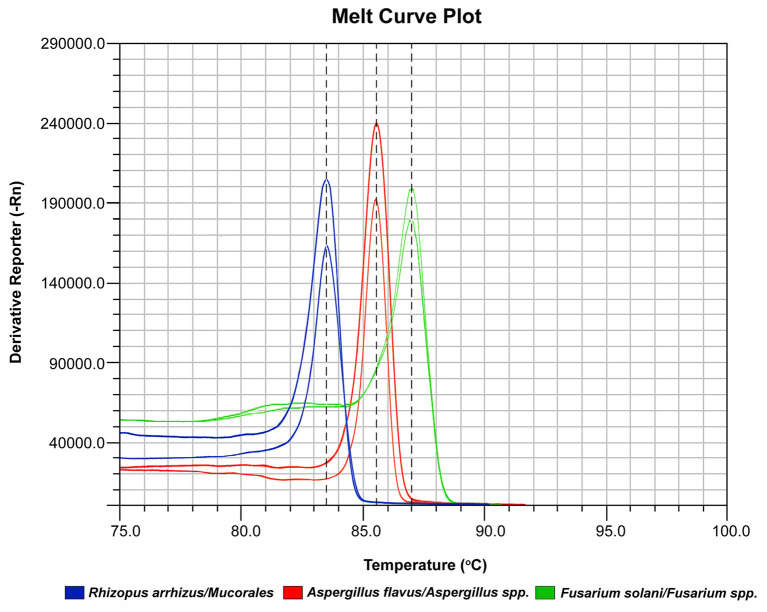
Variation in melt curve profile of different molds.

The melting temperature of the amplicons was determined as 85.5°C for *Aspergillus spp*, 83.5°C for *Mucorales*, and 87°C for *Fusarium spp*. (Figure 2).

**Figure 3:**
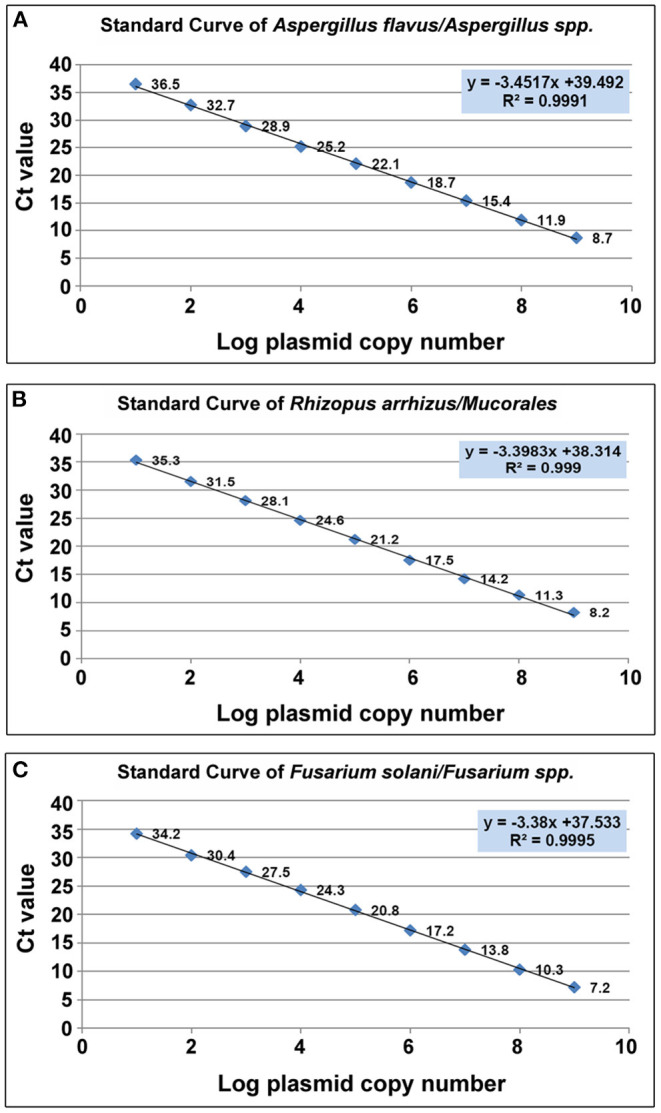
Scatter plot of CT value vis Log plasmid copy number. **(A)** *Aspergillus spp (A.flavus)*. **(B)** *Mucorales. (R.arrhizus)* **(C)** *Fusarium spp*. *(F.solani)*.

The cycle threshold (C_t_) values of individual standards were plotted against their respective plasmid DNA copy number, to produce a standard curve for each of the three target molds (Figure 3).which was then used to determine the sensitivity, primer efficiency and Limit of Detection (LoD) of the assay. The LoD was determined as 10 copies/μl for all three target molds, corresponding to C_t_ values of 36.5, 35.3, and 34.2 for *Aspergillus spp., Mucorales* and *Fusarium spp*. respectively.

Inter-assay variability was measured using the results of three separate assays performed on three different days, and intra-assay variability was measured by performing a single assay using each standard in quintuplicate. The inter-assay and intra-assay coefficient of variation (CV) was determined for each of the three target molds (*Aspergillus flavus, Rhizopus arrhizus, Fusarium solani*—Supplementary Table 6). No amplification was seen with human DNA or in any non-template controls (NTC). Inter-assay CV was <1.56% and intra-assay CV was <1% for all standards and all three targeted molds, demonstrating satisfactory repeatability, and reproducibility of the assay.

### Evaluation of Assay in Clinical Samples

A total of 150 cases of hematological malignancy suspected to have invasive aspergillosis (IA) and 239 cases of uncontrolled diabetes mellitus suspected to have invasive mucormycosis (IM) were recruited, and collection of clinical, radiological data and conventional laboratory diagnosis was conducted prospectively between April 2015 and May 2018. Molecular diagnosis was retrospective and non-interventional. Clinical categorization of cases was done according to EORTC/MSGERC guidelines (Donnelly et al., 2019). Details of case categorization and sample collection are given in Supplementary Table 3 and complete demographic details are given in Supplementary Tables 4, 5.

### Conventional Diagnosis

For cases of suspected IA, automated blood culture was done in BD BACTEC Peds Plus™ for pediatric patients and BD BACTEC Myco/F Lytic Medium for adult patients, incubated for a maximum duration of 2 weeks. Sub-cultures on Sabouraud's dextrose agar (SDA) for further identification were done from bottles which signaled positive. Galactomannan antigen assay was performed on serum samples using the Platelia™ *Aspergillus* Ag kit (Biorad, California, USA) following the manufacturer's instructions.

For cases of suspected IM, samples were subjected to direct microscopy of wet mounts (using KOH-calcofluor stain and an epifluorescence microscope), cultured on SDA slants and incubated at 25°C and 37°C for a maximum duration of 2 weeks. Mold isolates were further identified by colony morphology and microscopic appearance in lactophenol cotton blue (LPCB) mounts.

#### DNA Extraction

DNA extraction was done directly from clinical samples in a BSL-2 biological safety cabinet.

DNA extraction from EDTA whole blood from cases of suspected IA was performed using GSure Blood Mini-Kit (GCC Biotech, Kolkata, India) following the manufacturer's instructions. Positive controls (pooled blood from healthy volunteers spiked with ~100 spores of *A. fumigatus* per 500 μl of blood) and negative controls (pooled blood from healthy volunteers) were included in every extraction.

DNA extraction from tissue samples, BAL and other site-specific samples from cases of suspected IM was performed using the QIAamp tissue DNA extraction kit (Qiagen, Hilden, Germany). Samples were homogenized as described by Bialek et al. with a few modifications, in order to lyse the fungal cell wall and release fungal DNA (40). 140 μl of ATL buffer and 20 μl of proteinase K were added to 50–100 mg of tissue or 300 μl of liquid sample (BAL, pleural fluid etc), and incubated overnight at 56°C. The tubes were then placed in a boiling water bath at 100°C for 5 mins. A “freeze-and-thaw” step (consisting of incubation in liquid nitrogen for 1 min, followed by incubation in boiling water for 2 mins) was repeated 5 times. These homogenized samples were subjected to the remaining steps of the DNA extraction process according to the manufacturer's instructions. Since tissue samples could not be spiked to use as positive controls, conventional PCR targeting human beta globin gene was run for DNA extracted from all tissue samples, to check efficiency of extraction.

### Conventional PCR

For cases of suspected IA, DNA extracted from EDTA whole blood samples was subjected to pan-fungal PCR and *Aspergillus*-specific PCR as described in previous studies (White et al., 1990; Sugita et al., 2004; Muñoz-Cadavid et al., 2010; Rampini et al., 2016). For cases of suspected IM, DNA extracted from samples was subjected to panfungal PCR followed by sequencing of amplicons, and a semi-nested *Mucorales*-specific PCR as described in previous studies (White et al., 1990; Bialek et al., 2005; Muñoz-Cadavid et al., 2010; Rampini et al., 2016).

#### Real-Time PCR Under Evaluation in This Study

DNA extracted from EDTA whole blood samples for cases of suspected IA, and from tissue, BAL and other site-specific samples from cases of suspected IM, was subjected to quantitative real-time PCR and melting curve analysis as standardized above.

#### ROC Curve Generation

ROC curves were generated to calculate the cut-off copy number of target sequence considered significant. The gold standards were defined as follows:

- For cases of suspected IA: Presence of any one radiological finding suggestive of invasive pulmonary aspergillosis and positive result in galactomannan assay for two consecutive serum samples, both parameters defined according to EORTC guidelines (Donnelly et al., 2019).
- For cases of suspected IM: Presence of broad aseptate ribbon-like hyphae in tissue samples, detected in wet mounts using KOH-calcofluor stain and epifluorescence microscopy (independent of culture positivity, owing to well-documented poor recovery rates of *Mucorales* in culture even in proven cases).

### Statistical Analysis

All the statistical analysis was performed by Stata software, (Version 12). MedCalc (medcalc.org, Belgium) software was used to generate the ROC curves.

## Results and Discussion

This assay was evaluated in 2 groups of patients using various samples (whole blood in suspected IA and site-specific tissue and fluid samples in suspected IM). Sensitivity and specificity of real-time PCR was better than standard techniques like culture and conventional PCR in both groups of patients.

### Evaluation of Real-Time PCR in Cases of Suspected Invasive Aspergillosis (IA)

53 (35.3%) of the 150 IA cases were categorized as “probable,” 50 (33.3%) as “possible” and the remaining 47 as “no IA” according to EORTC guidelines (Donnelly et al., 2019), on the basis of clinical and radiological findings, positive galactomannan assay and conventional PCR positivity.

#### Validation of the Assay in Clinical Samples

**Figure 4:**
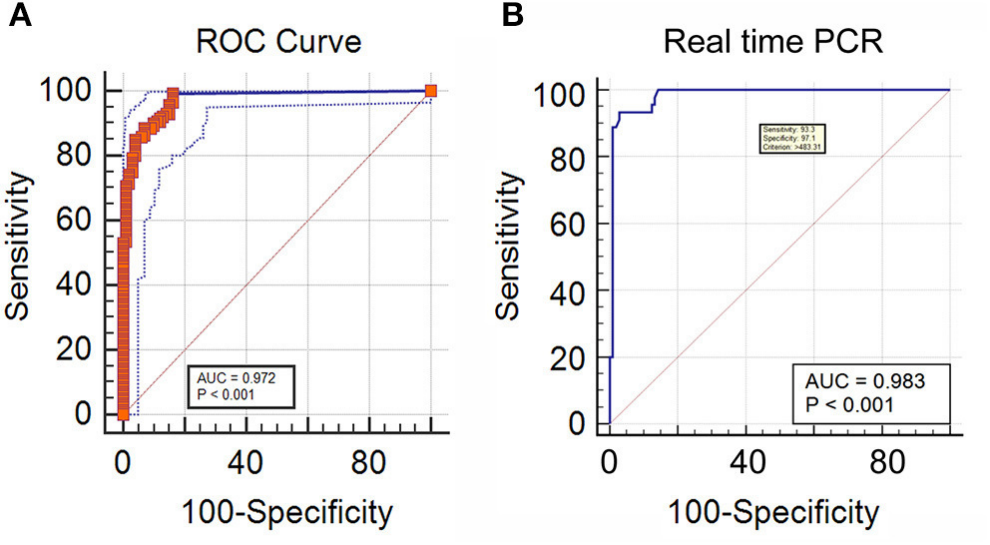
**(A)** Receiver operator curve plotted significant DNA copy number cut-off for IA cases, classified according to two consecutive GM positivity and radiology findings **(B)** ROC curve of real time PCR DNA copy number considering direct microscopy as a reference for IM.

In DNA extracted from EDTA blood from cases of IA, the real-time PCR was evaluated in comparison to a “gold standard” [Positive galactomannan assay results (GM index ≥ 1.0) in any two consecutive serum samples, along with radiological findings indicative of IA according to EORTC guidelines] for generation of an ROC curve to determine a significant cut off value for copy number of target sequence in samples from cases of suspected IA. This was determined to be 483.31 copies/ml of blood. with an area under the curve (AUC) of 0.983 (Figure 4A), Applying this cutoff, qPCR under evaluation showed 93.3% sensitivity, 97.1% specificity, 97.14% negative predictive value (NPV) and 93.33% positive predictive value (PPV) in detection of IA in suspected cases (Table 1). Using this cut-off, 65/150 patients (43.3%) showed positive PCR on day 0, 46/150 (30.6%) on day 7 and 15/150 (10%) on day 14. Sensitivity, specificity, PPV and NPV in probable and possible cases separately is given in Table 2. All the positive samples detected in real-time PCR were identified as *Aspergillus spp* by melting curve analysis.

**Table 1:** Sensitivity and specificity of real time PCR and conventional PCR taking positive radiological findings and two consecutive GM positivity as a reference.

	**Two consecutive GM positive** **+** **Positive radiology**			
	**Sensitivity %**	**Specificity %**	**PPV %**	**NPV %**
Conventional PCR	77.78	91.43	79.55	90.57
Real-time PCR	93.3	97.1	93.33	97.14

**Table 2:** Sensitivity, specificity, PPV, and NPV in probable and possible IA cases.

	**Sensitivity (%)**	**Specificity (%)**	**PPV (%)**	**NPV (%)**
**Probable cases**				
Real-time PCR	90.57	91.49	92.31	89.58
Conventional PCR	80	91.49	90.91	81.13
**Possible cases**				
Real-time PCR	34	91.49	80.95	56.58
Conventional PCR	14	91.49	63.64	50

In comparison, conventional PCR showed sensitivity and specificity of 77.78% and 91.43% respectively. Sensitivity and specificity of qPCR and conventional PCR are compared in Table 1.

The fungal load in blood of IA patients is thought to be very low (30–100 fg/μl or less) (Costa et al., 2002; Pham et al., 2003). Therefore high sensitivity is desirable in any assay to detect the presence of *Aspergillus spp* in blood. The specificity of the qPCR was comparable with other studies in which blood samples from hematological malignancy patients were tested by molecular methods for detection of *Aspergillus spp*, whereas its sensitivity was higher than reported in these studies (Kami et al., 2001). Other studies, in which PCR for detection of *Aspergillus spp* was performed on BAL samples from hematological malignancy patients, showed sensitivity of 88–90% and specificity of 95–100% (Sanguinetti et al., 2003; Imbert et al., 2018). However, BAL samples could not be included in the present study owing to the difficulty and risk involved in collecting BAL samples from critically ill patients.

The combination of galactomannan assay (performed on serum samples collected on Day 0) and the real-time PCR under evaluation was shown to be 100% specific for the diagnosis of probable cases of IA in this study, i.e., neither biomarker was positive in cases that did not meet the gold standard for comparison described earlier. A similar “combined biomarker” approach has been shown to be successful in other studies also (Botterel et al., 2008).

Automated blood culture showed no growth of fungi in all suspected cases of IA, which is concordant with similar low sensitivity of blood culture reported by Simoneau et al. (2005) for invasive aspergillosis in high-risk individuals. Therefore it appears to have limited utility even in conjunction with the real-time PCR.

Overall, 33.9% mortality was observed in probable cases of IA in this study, which was lower than the 50–100% mortality observed in other studies (Cornet et al., 2002; Meersseman et al., 2007). No significant difference in mortality between possible and probable cases was observed. Early death (within 20 days of admission to hospital) was observed in our study in IA patients with >8,000 copies of DNA per ml of blood, but there were too few cases in this category to establish an association between fungal load and mortality. Some previous studies evaluating this association reported significantly higher mortality in immunocompromised patients with >500 copies/ml BAL samples and >150 copies/ml of blood samples respectively with different group of immunocompromised patients (Imbert et al., 2016, 2018), while others found no significant association between clinical outcome and detection of *Aspergillus* DNA in clinical samples (Bergeron et al., 2012).

### Evaluation of Real-Time PCR in Cases of Suspected Invasive Mucormycosis (IM)

11 of the 239 suspected IM cases were categorized as “proven,” 129 as “probable,” and 17 as “possible” IM, while the remaining 82 cases were classified as “no IM,” according to EORTC guidelines (Donnelly et al., 2019) on the basis of clinical and radiological findings, direct microscopy and culture.

#### Validation of the Assay in Clinical Samples

In DNA extracted from tissue or other site-specific samples from cases of IM, The real-time PCR was evaluated in comparison to a “gold standard” of presence of broad aseptate ribbon-like hyphae in tissue or other site-specific samples using epifluorescence microscopy, for generation of an ROC curve to determine a significant cutoff value for copy number of target sequence in samples from cases of suspected IM (see Methodology). This was determined as ≥ 1 copy/ml, as detection of even a single copy of the target sequence in samples from cases of suspected IM was found to be significant. with area under the curve (AUC) of 0.972 (Figure 4B) and 99.29% sensitivity, 83.84% specificity, 98.81% negative predictive value (NPV) and 89.68% positive predictive value (PPV) in detection of IM in suspected cases (Table 3). This was mostly concordant with the results of previous studies on detection of Mucorales *via* multiplex real-time PCR in tissue samples (Bernal-Martinez et al., 2013), but showed a higher sensitivity (99.22%) in probable cases (Springer et al., 2016a).

**Table 3:** Diagnostic performance of molecular techniques in diagnosis of IM.

		**Sensitivity**	**Specificity**	**NPV**	**PPV**	**Concordance**
		**(%)**	**(%)**	**(%)**	**(%)**	**Rate**
**Performance in proven and probable IM (Direct microscopy as reference)**							
Proven cases (n = 11)	qPCR	100	83.84	100	40.74	85.45
	Panfungal PCR	100	73.74	100	29.73	76.36
	*Mucorales* specific PCR	100	91.92	100	57.89	92.73
Probable cases (*n* = 129)	qPCR	99.22	83.84	98.81	88.89	92.54
	Panfungal PCR	98.45	73.74	97.33	83.01	87.72
	*Mucorales* specific PCR	98.45	91.92	97.85	94.07	95.61
Overall	qPCR	99.29	83.84	98.81	89.68	92.89
	Panfungal PCR	98.57	73.74	97.33	84.15	88.28
	*Mucorales* specific PCR	98.57	91.92	97.85	94.52	95.82
**Performance in possible IM (Clinical and radiological findings as reference)**						
Possible cases (*n* = 17)	qPCR	82.35	97.56	96.39	87.50	94.95
	Panfungal PCR	70.59	82.93	93.15	46.15	80.81
	*Mucorales* specific PCR	47.06	100	90.11	100	90.91

A positive qPCR was seen in 11/11 proven cases, 128/129 probable cases, and 14/17 possible cases of IM, defined according to EORTC guidelines. All the positive samples detected in real-time PCR in proven and probable IM were identified as *Mucorales* by melting curveanalysis.

The real-time PCR evaluated in this study was also able to detect 14 out of 17 cases of possible invasive fungal sinusitis, defined according to EORTC guidelines (Donnelly et al., 2019), in which samples did not show fungal elements in direct epifluorescence microscopy or growth of fungi in culture, but clinical and radiological features of fungal sinusitis were observed. By use of melting curve analysis to differentiate *Mucorales* from *Aspergillus spp*, 10/14 cases were identified as possible invasive rhino-orbito-cerebral mucormycosis, and the remaining 4/14 were identified as possible fungal rhinosinusitis due to *Aspergillus spp*. Hence the real-time PCR is useful not only in detecting possible cases of invasive fungal sinusitis which might be missed by conventional methods of diagnosis, but also in differentiating possible rhino-orbito-cerebral mucormycosis from possible fungal rhinosinusitis due to *Aspergillus spp*, where this differentiation is not possible by other means, which has a significant impact on decisions regarding appropriate medical and surgical management of these cases.

**Figure 5:**
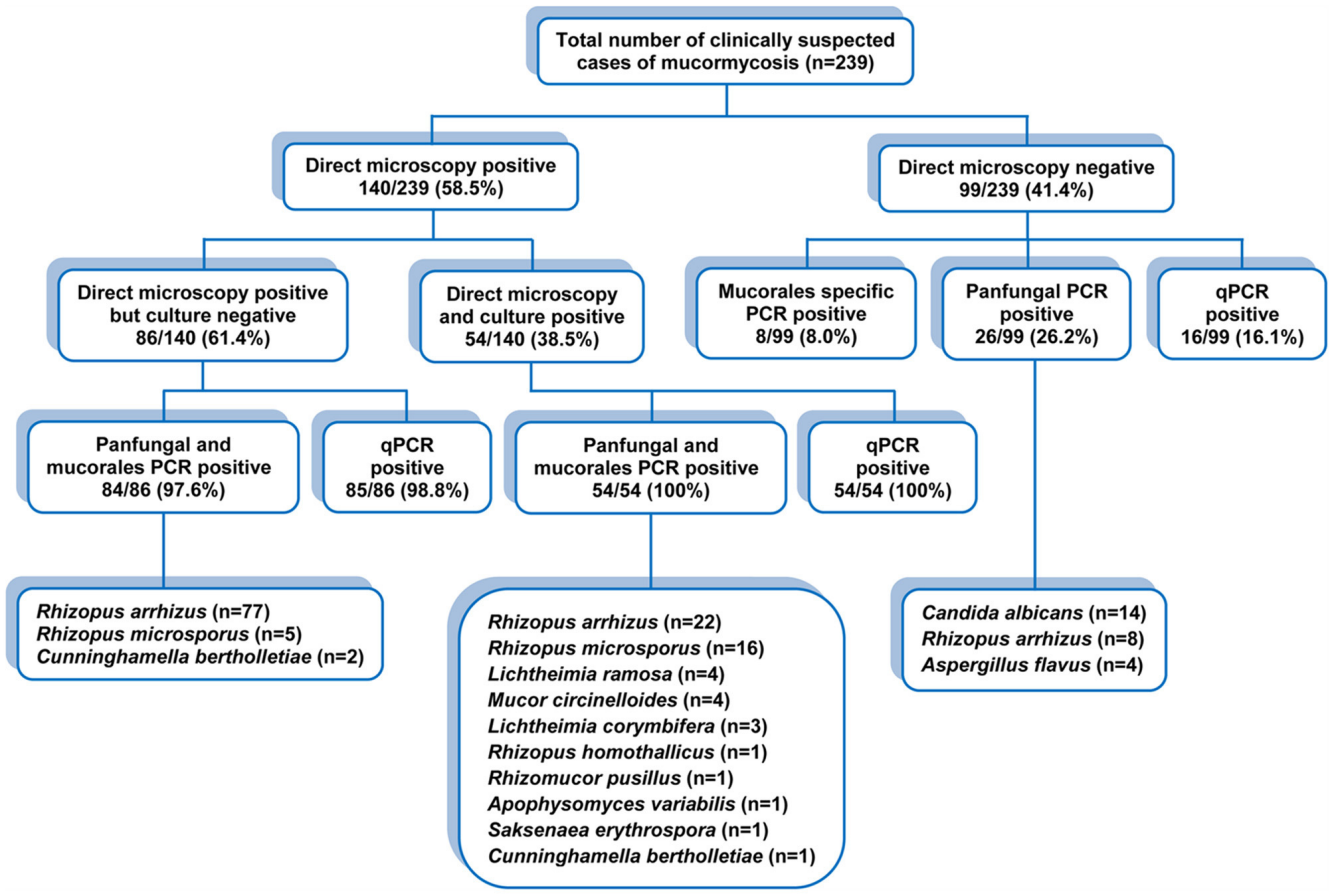
Detailed description of the results of conventional and molecular techniques (IM cases).

The performances of conventional panfungal PCR, conventional *Mucorales*-specific PCR and qPCR in diagnosis of proven, probable and possible IM are compared separately in Table 3 and Figure 5 depicts a detailed description of the results of conventional and molecular techniques (IM cases).

Since direct microscopy was used as the gold standard in generation of the ROC curve for the qPCR, an independent standard of comparison had to be established in order to compare conventional diagnosis (microscopy and culture) to molecular methods (conventional and qPCR). This independent standard was defined as a combination of clinical and radiological features suggestive of IM, along with response to antifungal treatment with liposomal amphotericin B. The comparison is shown in Table 4. Sensitivity and specificity of the real-time PCR were found to be 97.45 and 97.56% respectively. In comparison, the other diagnostic methods used in this study showed sensitivity and specificity of 89.17, and 100% (direct epifluorescence microscopy from samples), 34.39% and 100% (culture), 95.54 and 82.93% (panfungal PCR followed by sequencing of amplicons) and 92.9 and 100% (*Mucorales*-specific PCR), respectively.

**Table 4:** Comparison of sensitivity and specificity of qPCR with microscopy, culture, and conventional PCR in IM cases.

	**Clinical data radiological findings and response to antifungal treatment**			**Sensitivity %**	**Specificity %**	**NPV %**	**PPV %**
		**Positive (*n* = 157)**	**Negative (*n* = 82)**				
Real time PCR	Positive	153	2	97.45	97.56	95.24	98.71
	Negative	4	80				
Panfungal PCR	Positive	150	14	95.54	82.93	90.67	91.46
	Negative	7	68				
*Mucorales* specific PCR	Positive	146	0	92.9	100	88.17	100
	Negative	11	82				
Direct Microscopy	Positive	140	0	89.17	100	82.83	100
	Negative	17	82				
Culture	Positive	54	0	34.39	100	44.32	100
	Negative	103	82				

It is evident that the real-time PCR showed higher sensitivity than all the other diagnostic methods, and also a higher specificity than all methods except culture. However the sensitivity of culture is the lowest out of all the methods, thus limiting its utility overall as a reliable method of diagnosis. This is in concordance with previous studies (Chakrabarti et al., 2006; Richardson and Lass-Flörl, 2008; Prakash et al., 2019), especially in diagnosis of pulmonary mucormycosis (Lengerova et al., 2014; Scherer et al., 2018), although some studies reported higher sensitivity and lower specificity of culture than observed in the present study (Badiee et al., 2013).

Early mortality (within 30 days) was observed in IM cases with >10,000 DNA copies/ mg of tissue. However, as observed previously in patients with IA, these cases were too few to establish a definite association between fungal load and mortality.

Finally, 15 out of 17 cases of co-infection (both IM and aspergillosis, in which both septate and aseptate hyphae were seen in samples in direct microscopy) were detected by real-time PCR among the cases of suspected IM, and differentiated by melting curve analysis (Only *Mucorales* was detected by melting curve analysis in the remaining 2/17). Detailed descriptions of these cases of co-infection are given in Supplementary Table 7. Since the sample in all these cases was tissue from paranasal sinuses, it is impossible to comment on whether the detection of *Aspergillus spp* indicates only colonization or might be contributing to invasive sinusitis along with *Mucorale*s, according to EORTC guidelines. However, this ability of the assay could prove useful in detection of fungal co-infections in samples such as blood and tissue/fluids from sterile sites.

## Limitations

*Fusarium spp* was not detected in any clinical samples; therefore the utility of this assay in diagnosis of fusariosis remains to be evaluated. Neither was it evaluated using respiratory samples from cases of suspected invasive aspergillosis, owing to the risk and difficulty involved in collecting respiratory samples from patients with hematological malignancies, who were already critically ill and immunosuppressed, therefore were not subjected to invasive diagnostic procedures such as broncho-alveolar lavage. Since this was a newly developed assay under evaluation, the results were not used to guide clinical intervention and its impact on outcome could not be assessed.

## Conclusion

The real-time PCR assay using newly designed primers evaluated in this study is superior to standard conventional methods of diagnosis and is a fast, sensitive, and specific method for the diagnosis of invasive mold infections. It can detect and differentiate all three clinically relevant molds prevalent in this geographical area, without the need for amplicon sequencing, this would be especially useful in cases of hematological malignancy, who can be affected by invasive fungal infections due to all three molds, and in detection of cases of mixed infection. Used in combination with the galactomannan assay, it can confirm the diagnosis of invasive aspergillosis in high-risk patients with 100% specificity. Due to its ability to detect possible cases of invasive mucormycosis which are missed by conventional methods, it would also be useful in early diagnosis and treatment for better clinical outcomes, or in detecting cases which have relapsed after incomplete treatment. Further studies are needed to expand the melting curve database and increase the number of fungal species that can be identified by melting curve analysis.

## Data Availability Statement

The datasets presented in this study can be found in online repositories. The names of the repository/repositories and accession number(s) can be found in the article/Supplementary Material.

## Ethics Statement

The study was conducted on patient's samples after receiving written informed consent and the study was approved by the Ethical Committee of the All India Institute of Medical Sciences (No- IESC/T-13/01.04.2015). Written informed consent to participate in this study was provided by the participants' legal guardian/next of kin.

## Author Contributions

MP: carried out the majority of the experiments for this study, experimental design, data analysis, interpretation, and writing of the paper. IX: design of the study, interpretation of data for the work, data editing and revising it critically for important intellectual content, procured funding and resources in order to complete the work, and final approval of the version to be published. JS: critical revision of the article. UY: greatly helped in the cloning experiment for the generation of standard curves. GS: conventional diagnosis of the clinical isolates, helped in data analysis, and contributed analysis tool. DP and AJ: contributed to the designing of the primer for the study. AX: helped with data collection and conventional diagnosis. BR: preserved the culture isolates used to evaluate the specificity of the primers. LD: greatly aided in the research methodology and the selection of patient populations. SB, RS, MM, VJ, and RK: aided in patient recruitment and provided clinical samples. RA: maintained the clinical isolates and assisted in the writing of the paper. PM: formatted tables and figures in the manuscript in accordance with journal guidelines. All authors contributed to the article and approved the submitted version.

## Conflict of Interest

The authors declare that the research was conducted in the absence of any commercial or financial relationships that could be construed as a potential conflict of interest.

## Publisher's Note

All claims expressed in this article are solely those of the authors and do not necessarily represent those of their affiliated organizations, or those of the publisher, the editors and the reviewers. Any product that may be evaluated in this article, or claim that may be made by its manufacturer, is not guaranteed or endorsed by the publisher.

## Supplementary Material

Supplementary Figure 1 Agarose gel electrophoresis image of PCR products of newly designed mold specific primer set.

Supplementary Table 1 Fungi used to check the specificity of new qPCR assay.

Supplementary Table 2 Accession numbers of sequences downloaded from the GenBank and strains used for the validation of technique.

Supplementary Table 3 Categorization of cases as per EORTC/MSGERC guidelines.

Supplementary Table 4 Demographic details of suspected IA patients.

Supplementary Table 5 Demographic and clinical characteristics of suspected IM patients.

Supplementary Table 6 Repeatability and reproducibility of qPCR assay for different molds.

Supplementary Table 7 Detailed description of co-infection cases.
