# Supplementary figures and images for: Development of a Sensitive and Specific Novel qPCR Assay for Simultaneous Detection and Differentiation of Mucormycosis and Aspergillosis by Melting Curve Analysis
Source: Front Fungal Biol. 2022 Jan 24;2:800898. doi: 10.3389/ffunb.2021.800898 (PMC10512281; doi:10.3389/ffunb.2021.800898)

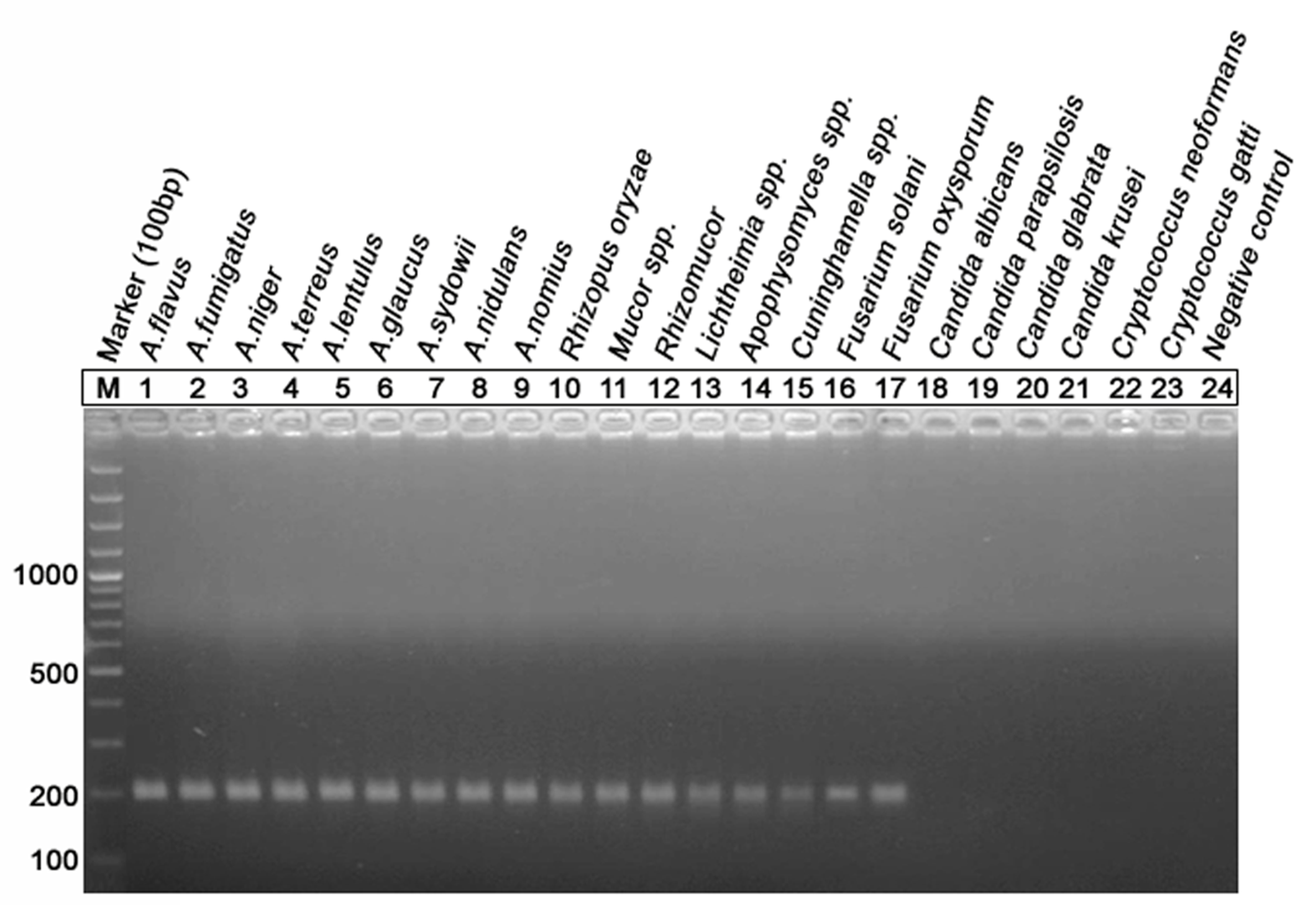

Supplement: Supplementary Figure 1 — Agarose gel electrophoresis image of PCR products of newly designed mold specific primer set. [file Image_1.TIF]
