# Supplementary material for: Development of a Sensitive and Specific Novel qPCR Assay for Simultaneous Detection and Differentiation of Mucormycosis and Aspergillosis by Melting Curve Analysis
Source: Front Fungal Biol. 2022 Jan 24;2:800898. doi: 10.3389/ffunb.2021.800898 (PMC10512281; doi:10.3389/ffunb.2021.800898)
Supplement: Supplementary Table 1 — Fungi used to check the specificity of new qPCR assay. [file Table_1.DOCX]

**Supplementary Material**

**Table S1: Fungi used to check the specificity of new qPCR assay**

| ***Mucorales Fungi*** | **Amplification** | ***Aspergillus sp.*** | **Amplification** |
| --- | --- | --- | --- |
| *R.oryzae* | Yes | *A. flavus* | Yes |
| *R.microsporus* | Yes | *A.fumigatus* | Yes |
| *Mucor circinelloides* | Yes | *A.niger* | Yes |
| *Lichthiemia corymbifera* | Yes | *A.terreus* | Yes |
| *Cunninghamella bertholletiae* | Yes | *A.nidulans* | Yes |
| *Saksenaea erythrospora* | Yes | *A.glaucus* | Yes |
| *Apophysomyces variabilis* | Yes | *A.sydowii* | Yes |
| *Rhizomucor pusillus* | Yes | *A.nomius* | Yes |
|  |  | *A.lentulus* | Yes |
| **Dermatophytes** | **Amplification** |  |  |
| *Trichophyton rubrum* | *Yes* | ***Yeasts*** | **Amplification** |
| *Trichophyton tonsurans* | *Yes* | *Candida albicans* | *No* |
| *Trichophyton mentagrophytes* | *Yes* | *Candida tropicalis* | *No* |
| *Microsporum spp.* | *Yes* | *Candida parapsilosis* | *No* |
| ***Fusarium sp.*** | **Amplification** | *Candida glabrata* | *No* |
| *Fusarium solani* | *Yes* | *Candida dubliniensis* | *No* |
| *Fusarium oxysporum* | *Yes* | *Candida krusei* | *No* |
|  |  | *Candida auris* | *No* |
| **Fungi** | **Amplification** | *Cryptococcus neoformans* | *No* |
| *Histoplasma spp* | *No* | *Cryptococcus gatti* | *No* |
| *Sporothrix* | *No* | *Trichosporonsp.* | *No* |
| *Talaromyces marneffei* | *No* | **Other Fungi** | **Amplification** |
| *Acremonium* | *No* | *Cladophialophorabantiana* | *No* |
| *Schizophyllum* | *No* | *Exophiala spinifera* | *No* |
| *Alternaria* | *No* | *Cladosporium* | *No* |
| *Bipolaris* | *No* |  |  |
| *Scopulariopsis* | *No* | *Alternaria spp* | *No* |
| *Fonsecaea pedrosoi* | *No* |  |  |

**Table S2: Accession numbers of sequences downloaded from the GenBank and strains used for the validation of technique**

| **Accession numbers of the sequences downloaded from the GenBank** | |
| --- | --- |
| **Strain** | **Accession Number** |
| *Rhizopus sp.* | EF623861 |
| *Rhizopus arrhizus* | GU594768 |
| *Rhizopus microsporus* | GQ502280 |
| *Mucor racemosus* | JF440624 |
| *Rhizomucor pusillus* | MN386256 |
| *Mucor circinelloides* | JX537952 |
| *Lichtheimia corymbifera* | JX537953 |
| *Apophysomyces elegans* | NR_149336 |
| *Cunninghamella bertholletiae* | JN205879 |
| *Aspergillus flavus* | GU594737 |
| *Aspergillus fumigatus* | MG461649 |
| *Aspergillus niger* | MT620753 |
| *Aspergillus terreus* | KC113303 |
| *Aspergillus nidulans* | KR150743 |
| *Aspergillus sydowii* | EF652451 |
| *Aspergillus sp.* | MG807064 |
| *Aspergillus lentulus* | KX903293 |
| *Fusarium solani* | MN857748 |
| *Fusarium oxysporum* | KU872838 |
| **Accession numbers of the sequences of the isolates used in this study** | |
| *Rhizopus arrhizus* | OL662892 |
| *Rhizopus microsporus* | MG972786 |
| *Mucor circinelloides* | OL662891 |
| *Rhizomucor pusillus* | OL672705 |
| *Lichtheimia corymbifera* | MK411419 |
| *Apophysomyces variabilis* | OL662914 |
| *Cunninghamella bertholletiae* | OL672664 |
| *Aspergillus flavus* | OL662888 |
| *Aspergillus fumigatus* | MK411417 |
| *Aspergillus niger* | OL662886 |
| *Aspergillus terreus* | OL662887 |
| *Aspergillus nidulans* | MK425749 |
| *Aspergillus sydowii* | MK425750 |
| *Aspergillus lentulus* | MK621455 |
| *Fusarium solani* | OL687415 |
| *Fusarium oxysporum* | OL662893 |

**Table S3: Categorization of cases as per EORTC/MSGERC guidelines**:

|  | **Suspected cases of invasive aspergillosis**  **(n=150)** | **Suspected cases of invasive mucormycosis (n=239)** |
| --- | --- | --- |
| **Proven** | 0 | 11 (4.6%) |
| **Probable** | 53 (35.3%) | 129 (53.9%) |
| **Possible** | 50 (33.3%) | 17 (7.1%) |
| **No IFI** | 47 (31.3%) | 82 (34.3%) |
| **Radiology** | n=150 | n=239 |
|  | Ground Glass Opacity (GGO) (n=29) | ROCM cases with  sino-nasal involvement (n=29) |
|  | Nodule with GGO(n=41) | ROCM cases with  sino-orbital involvement (n=63) |
|  | Consolidation (n=11) | ROCM cases with intracranial extension (n=40) |
|  | halo sign (n=13) | Pulmonary mucormycosis (n=23) |
|  | Normal findings (n=41) | Normal Findings (n=48) |
|  | Not done (n=6) | Not Done (n=36) |
| **Samples** | 3 samples collected from each patient on Day 0, Day 7, Day 14 (Total 450 samples of each type)   - BACTEC blood (For culture ) - Serum (For galactomannan Ag test) - EDTA whole blood (For molecular tests) | Sinus Biopsy (n=171)  BAL/mini-BAL (n=21)  Nasal Crust (n=21)  Orbital Tissue (n=9)  Palatal Tissue (n=9)  Lung Biopsy (n=2)  Nasal Discharge (n=2)  Nasal Swab (n=1)  Pleural Fluid (n=1)  Skin Biopsy/Abdominal wound tissue (n=2) |

**Table S4: Demographic details of suspected IA patients**

| **Characteristics** | **Variable** | **Value** | **Expired** | **Improved** | **p-value Mortality** | **Total** |
| --- | --- | --- | --- | --- | --- | --- |
| **Gender** | Male | 99 | 35 | 64 | 0.48 | 150 |
|  | Female | 51 | 21 | 30 |  |  |
| **Age** | Median Years | 24 |  |  |  |  |
|  | Mean | 26.76 |  |  |  |  |
|  | 0-5 | 21 | 5 (23.8 %) | 16 (76.2 %) | 0.498 | 150 |
|  | 6-17 | 27 | 9 (33.3 %) | 18 (66.7 %) |  |  |
|  | 18-44 | 75 | 32 (44 %) | 42 (56 %) |  |  |
|  | 45-65 | 27 | 10 (37 %) | 17 (63 %) |  |  |
|  | >65 | 1 | 0 (0 %) | 1 (100 %) |  |  |
| **Malignancy** | AML | 95 | 38 (40 %) | 57 (60 %) | 0.39 | 150 |
|  | ALL | 55 | 18 (32.7 %) | 37 (67.3 %) |  |  |

| **Characteristic** | **Variable** | **Value** | **DM**  **(n=213)** | **DM with ketoacidosis (n=4)** | **Hematological Malignancy**  **(n=11)** | | | | | **DM with CKD**  **(n=6)** | **CKD**  **(n=3)** | **No Risk factor**  **(n=2)** |
| --- | --- | --- | --- | --- | --- | --- | --- | --- | --- | --- | --- | --- |
|  |  |  |  |  | **AML** | **ALL** | **CML** | **Aplastic Anemia** | **Lymphoma** |  |  |  |
| Gender | Male | 161 | 141 | 2 | 3 | 3 | 2 | 1 | 1 | 5 | 1 | 2 |
|  | Female | 78 | 72 | 2 | 0 | 0 | 0 | 0 | 1 | 1 | 2 | 0 |
| Age (years)  (median age= 41years) | 0-17 | 18 | 16 | 0 | 0 | 1 | 0 | 0 | 0 | 0 | 0 | 1 |
|  | 18-65 | 208 | 185 | 4 | 3 | 2 | 2 | 1 | 2 | 5 | 3 | 1 |
|  | >65 | 13 | 12 | 0 | 0 | 0 | 0 | 0 | 0 | 1 | 0 | 0 |
| ROCM without  cerebral involvement | 65 Improved | 89 | 83 | 0 | 1 | 0 | 1 | 0 | 1 | 2 | 1 | 0 |
|  | 24 Expired |  |  |  |  |  |  |  |  |  |  |  |
| ROCM with cerebral involvement | 22 Improved | 40 | 32 | 3 | 2 | 0 | 0 | 0 | 1 | 1 | 0 | 1 |
|  | 18 Expired |  |  |  |  |  |  |  |  |  |  |  |
| Pulmonary mucormycosis | 11 Improved | 23 | 13 | 1 | 0 | 3 | 1 | 1 | 0 | 3 | 1 | 0 |
|  | 12 Expired |  |  |  |  |  |  |  |  |  |  |  |
| Cutaneous mucormycosis | 1 Improved | 2 | 1 | 0 | 0 | 0 | 0 | 0 | 0 | 0 | 0 | 1 |
|  | 1 Expired |  |  |  |  |  |  |  |  |  |  |  |
| Invasive  fungal sinusitis | 2 Improved | 3 | 3 | 0 | 0 | 0 | 0 | 0 | 0 | 0 | 0 | 0 |
|  | 1 Expired |  |  |  |  |  |  |  |  |  |  |  |
| No IFI | 74 Improved | 82 | 80 | 0 | 0 | 0 | 0 | 0 | 0 | 1 | 1 | 0 |
|  | 8 Expired |  |  |  |  |  |  |  |  |  |  |  |
| Two-months  mortality | Improved | 177 | 167 | 2 | 1 | 1 | 1 | 0 | 1 | 4 | 0 | 0 |
|  | Expired | 62 | 46 | 2 | 2 | 2 | 1 | 1 | 1 | 2 | 3 | 2 |
| Mortality  (Total) | Improved | 101 | 92 | 2 | 1 | 0 | 1 | 0 | 1 | 4 | 0 | 0 |
|  | Expired | 56 | 40 | 2 | 2 | 3 | 1 | 1 | 1 | 2 | 2 | 2 |

*Mortality was significantly higher in ROCM cases with cerebral involvement (p= 0.002). AML-Acute myeloid leukemia, ALL- Acute lymphocytic leukemia, CML- Chronic myeloid leukemia, DM- Diabetes mellitus, CKD- Chronic kidney disease

**Table S5: Demographic and clinical characteristics of suspected IM patients:**

**Table S6: Repeatability and reproducibility of qPCR assay for different molds**

|  | **Inter assay reproducibility** | | | **Intra assay reproducibility** | | |
| --- | --- | --- | --- | --- | --- | --- |
| **Plasmid Copy No.** | **Mean** | **Standard Deviation** | **Coefficient of Variation (%)** | **Mean** | **Standard Deviation** | **Coefficient of Variation(%)** |
| ***Aspergillus spp. (Aspergillus flavus)*** | | | | | | |
| 10^9^ | 8.72 | 0.12 | 1.42 | 8.66 | 0.07 | 0.82 |
| 10^8^ | 11.8 | 0.16 | 1.35 | 11.84 | 0.08 | 0.74 |
| 10^7^ | 15.4 | 0.20 | 1.30 | 15.54 | 0.11 | 0.73 |
| 10^6^ | 18.7 | 0.26 | 1.41 | 18.62 | 0.10 | 0.55 |
| 10^5^ | 22.2 | 0.29 | 1.31 | 22.33 | 0.20 | 0.90 |
| 10^4^ | 25.3 | 0.33 | 1.33 | 25.36 | 0.14 | 0.58 |
| 10^3^ | 28.7 | 0.32 | 1.12 | 28.82 | 0.21 | 0.72 |
| 10^2^ | 32.5 | 0.43 | 1.32 | 32.62 | 0.17 | 0.52 |
| 10 | 36.5 | 0.37 | 1.02 | 36.42 | 0.23 | 0.63 |
| **Inter assay mean CV: 1.29% Intra assay mean CV:0.69% Overall mean CV:0.99%** | | | | | | |
| ***Mucorales Fungi (Rhizopus arrhizus)*** | | | | | | |
| 10^9^ | 8.27 | 0.09 | 1.20 | 8.18 | 0.078 | 0.96 |
| 10^8^ | 11.15 | 0.16 | 1.44 | 11.21 | 0.09 | 0.82 |
| 10^7^ | 14.24 | 0.22 | 1.56 | 14.33 | 0.13 | 0.93 |
| 10^6^ | 17.71 | 0.19 | 1.11 | 17.61 | 0.10 | 0.57 |
| 10^5^ | 21.22 | 0.31 | 1.48 | 21.37 | 0.10 | 0.51 |
| 10^4^ | 24.59 | 0.37 | 1.52 | 24.72 | 0.15 | 0.64 |
| 10^3^ | 28.17 | 0.28 | 1.00 | 28.27 | 0.14 | 0.50 |
| 10^2^ | 31.55 | 0.34 | 1.07 | 31.54 | 0.25 | 0.80 |
| 10 | 35.31 | 0.40 | 1.14 | 35.63 | 0.32 | 0.90 |
| **Inter assay mean CV: 1.28% Intra assay mean CV:0.739% Overall mean CV:1.01%** | | | | | | |
| ***Fusarium spp. (Fusarium solani)*** | | | | | | |
| 10^9^ | 7.36 | 0.10 | 1.49 | 7.25 | 0.05 | 0.78 |
| 10^8^ | 10.33 | 0.15 | 1.52 | 10.33 | 0.08 | 0.78 |
| 10^7^ | 13.84 | 0.21 | 1.56 | 13.80 | 0.11 | 0.84 |
| 10^6^ | 17.28 | 0.25 | 1.49 | 17.36 | 0.10 | 0.59 |
| 10^5^ | 20.75 | 0.28 | 1.36 | 20.77 | 0.14 | 0.68 |
| 10^4^ | 24.35 | 0.27 | 1.11 | 24.35 | 0.23 | 0.95 |
| 10^3^ | 27.51 | 0.30 | 1.12 | 27.59 | 0.11 | 0.42 |
| 10^2^ | 30.45 | 0.39 | 1.29 | 30.53 | 0.14 | 0.46 |
| 10 | 34.48 | 0.47 | 1.37 | 34.20 | 0.15 | 0.44 |
| **Inter assay mean CV: 1.37% Intra assay mean CV:0.66% Overall mean CV:1.01%** | | | | | | |

**Table S7. Detailed description of co-infection cases**

|  | **Nasal tissue**  **(n=6)** | **BAL**  **(n=5)** | **Sinus tissue**  **(n=3)** | **Nasal crust**  **(n=2)** | **Abdominal wound pus tissue(n=1)** |
| --- | --- | --- | --- | --- | --- |
| **Direct Microscopy** | Septate+  aseptate hyphae  (n=6) | Septate+  aseptate hyphae  (n=5) | Septate+ aseptate hyphae (n=3) | Septate+ aseptate hyphae (n=2) | Septate+ aseptate hyphae (n=1) |
| **Culture** | *A. flavus* (n=3)  *A.fumigatus*(n=1)  Negative (n=2) | *A. flavus*+ *Lichtheimia spp*. (n=2)  *A. niger* + *Lichtheimia* *spp.*(n=1)  *R. microsporus*+  *A. flavus* (n=1)  *A. flavus* (n=1) | *A. flavus* (n=2)  *A. niger* (n=1) | *A. flavus* (n=2) | *R. arrhizus*+ *A. flavus* (n=1) |
| **Panfungal PCR** | Positive(n=6)  Negative(n=0) | Positive (n=5)  Negative (n=0) | Positive (n=3)  Negative (n=0) | Positive (n=2)  Negative (n=0) | Positive (n=1)  Negative (n=0) |
| **Mucorales specific PCR** | Positive (n=6)  Negative(n=0) | Positive (n=5)  Negative (n=0) | Positive (n=3)  Negative (n=0) | Positive (n=2)  Negative (n=0) | Positive (n=1)  Negative(n=0) |
| ***Aspergillus* specific PCR** | Positive (n=6) | Positive  (n=5)  Negative  (n=0) | Positive (n=3)  Negative (n=0) | Positive (n=2)  Negative (n=0) | Positive  (n=1)  Negative  (n=0) |
| ***Mucorales sp*. identified after panfungal PCR sequencing** | *R. arrhizus* (n=5)  *R. microsporus*  (n=1) | *Lichtheimia corymbifera* (n=2)  *Lichtheimia ramosa*(n=1) | *R. arrhizus*(n=3) | *R. arrhizus*(n=2) | *R. arrhizus* (n=1) |
| ***Mucorales* and *Aspergillus spp.* differentiated by qPCR melting curve analysis** | *Mucorales spp (n=6)*  *Aspergillus spp. (n=6)* | *Mucorales spp.(n=3)*  *Aspergillus spp(n=3)* | *Mucorales spp.(n=3)*  *Aspergillus spp(n=3)* | *Mucorales spp.(n=2)*  *Aspergillus spp(n=2)* | *Mucorales spp.(n=1)*  *Aspergillus spp(n=1)* |
